# Early medication use in new-onset rheumatoid arthritis may delay joint replacement: results of a large population-based study

**DOI:** 10.1186/s13075-015-0713-3

**Published:** 2015-08-03

**Authors:** Cristiano S. Moura, Michal Abrahamowicz, Marie-Eve Beauchamp, Diane Lacaille, Yishu Wang, Gilles Boire, Paul R. Fortin, Louis Bessette, Claire Bombardier, Jessica Widdifield, John G. Hanly, Debbie Feldman, Walter Maksymowych, Christine Peschken, Cheryl Barnabe, Steve Edworthy, Sasha Bernatsky, CAN-AIM

**Affiliations:** Department of Epidemiology, Biostatistics and Occupational Health, McGill University, Montreal, QC Canada; Division of Clinical Epidemiology, McGill University Health Centre, 687 Pine Avenue West, V-Building (V2.09), Montreal, QC H3A 1A1 Canada; Department of Medicine, University of British Columbia, Vancouver, BC Canada; Department of Medicine, Université de Sherbrooke, Sherbrooke, QC Canada; Division of Rheumatology, Department of Medicine, Université Laval, Quebec City, QC Canada; Division of Rheumatology, University of Toronto, Toronto, ON Canada; Institute for Clinical Evaluative Sciences, Toronto, ON Canada; Division of Rheumatology, Department of Medicine, Dalhousie University, Halifax, NS Canada; École de Réadaptation, Université de Montréal, Montreal, QC Canada; Department of Medicine, University of Alberta, Edmonton, AB Canada; Department of Internal Medicine, University of Manitoba, Winnipeg, MB Canada; Department of Medicine, University of Calgary, Calgary, AB Canada; Department of Community Health Sciences, University of Calgary, Calgary, AB Canada

## Abstract

**Introduction:**

Use of disease-modifying anti-rheumatic drugs (DMARDs) in rheumatoid arthritis (RA) may prevent joint damage and potentially reduce joint replacement surgeries. We assessed the association between RA drug use and joint replacement in Quebec, Canada.

**Methods:**

A cohort of new-onset RA patients was identified from Quebec’s physician billing and hospitalization databases from 2002–2011. The outcome was defined using procedure codes submitted by orthopedic surgeons. Medication use was obtained from pharmacy databases. We used alternative Cox regression models with time-dependent variables measuring the cumulative effects of past use during different time windows (one model focussing on the first year after cohort entry) for methotrexate (MTX), and other DMARDs. Models were adjusted for baseline sociodemographics, co-morbidity and prior health service use, time-dependent cumulative use of other drugs (anti-tumor necrosis factor [anti-TNF] agents, other biologics, cyclooxygenase-2 inhibitors [COXIBs], nonselective nonsteroidal antiinflammatory drugs [NSAIDs], and systemic steroids), and markers of disease severity.

**Results:**

During follow-up, 608 joint replacements occurred among 11,333 patients (median follow-up: 4.6 years). The best-fitting model relied on the cumulative early use (within the first year after cohort entry) of MTX and of other DMARDs, with an interaction between MTX and other DMARDs. In this model, greater exposure within the first year, to either MTX (adjusted hazard ratio, HR = 0.95 per 1 month, 95 % confidence interval, 95 % CI 0.93-0.97) or other DMARDs (HR = 0.97, 95 % CI 0.95-0.99) was associated with longer time to joint replacement.

**Conclusions:**

Our results suggest that longer exposure to either methotrexate (MTX) or other DMARDs within the first year after RA diagnosis is associated with longer time to joint replacement surgery.

**Electronic supplementary material:**

The online version of this article (doi:10.1186/s13075-015-0713-3) contains supplementary material, which is available to authorized users.

## Introduction

Rheumatoid arthritis (RA) is a serious inflammatory arthritis and affects 1 % of the population in the developed world [1]. RA is characterized by joint pain and swelling, which may result in physical impairments, joint deformity, disability, and decreased quality of life. The pain and loss of function in RA largely correlates with the extent of uncontrolled disease activity.

Treatment strategies for RA have improved dramatically over the past decade. Furthermore, earlier treatment increases the chance of clinical response and disease remission [2, 3] compared to the same treatment administered later in the disease course [4]. Disease-modifying anti-rheumatic drugs (DMARDs) include methotrexate (MTX), which is widely considered the cornerstone in RA care.

Uncontrolled RA activity can lead to irreversible joint damage requiring joint replacement surgery [5]. There are increasing randomized controlled (and observational) data suggesting that early and targeted treatment decreases joint damage in RA. It is hoped that prompt initiation of therapy and better control of disease could ultimately decrease the long-term need for orthopedic surgery. Population-based studies have demonstrated that orthopedic surgery rates have declined alongside the concomitant increase in anti-rheumatic drug use [6], however, it is uncertain to what extent early therapy ultimately reduces the need for later joint replacement surgery in RA.

In this study, we hypothesized that early drug exposure soon after RA diagnosis reduces the risk of subsequent joint replacement surgery. To assess this issue, we studied the potential association between RA drug use and joint replacement using a population-based RA cohort assembled from health administrative databases in Quebec.

## Methods

### Data sources

We performed cohort analyses on new-onset RA patients with public prescription drug coverage identified in the Quebec Health Insurance Program (RAMQ) databases from 2002–2011. In principle, as in each province in Canada, all Quebec residents (approximately 7.6 million persons) have access to comprehensive health care in terms of physician visits and hospitalization. Administrative databases record hospitalization and physician billing data for all residents of Quebec. The data include hospitalization discharge diagnoses (a primary diagnosis and up to 15 non-primary diagnoses per hospitalization, abstracted by medical records clerks), and physician-visit billing-claim diagnostic codes (a single diagnostic code is allowed per visit). Those residents who are beneficiaries of the provincial drug plan (which includes all seniors, and any non-seniors without private drug insurance, representing approximately 40 % of residents) can also be linked to the province’s dispensed prescription claims database (which includes information about drug, dose, duration, and dates).

We received ethics approval from the Quebec Commission for Access to Information. All data were de-nominalized, and identifying health card numbers were scrambled by the Quebec Health Insurance Board. As the data are anonymous, no informed consent is required from individual patients.

### Study population

To establish a population-based incident RA sample, all physician visits with an RA diagnosis code between 1 January 2002 and 31 December 2011 were identified. To increase the positive predictive value and specificity of the RA case definition, cases required at least three visit billing codes using International Classification of Diseases (ICD)-9, code 714 over a 3-year period, at least one of which was by an internist or a rheumatologist. This represents an adaptation of the RA diagnosis that was recently validated by Widdifield et al. (2013) using Ontario administrative data [7]. To identify truly incident (rather than prevalent) RA cases, we removed any patient who had any billing codes for RA prior to 1 January 2002, as data were available from 1989. Patients were followed from cohort entry (time when they fulfilled the RA case definition) until their first joint replacement surgery, or were censored at death date, or the end of study period (31 December 2011), whichever came first. We restricted our analyses to those patients who had drug coverage by the public drug program at the cohort entry and during at least 80 % of their follow-up time and who had follow up longer than 1 year.

### Exposure assessment

For each prescription of MTX, or other DMARDs (sulfasalazine, chloroquine, hydroxychloroquine, leflunomide, cyclosporine, minocycline, penicillamine, and cyclophosphamide), anti-TNF inhibitors, other biologic DMARDs (anakinra, rituximab, abatacept) cyclooxygenase-2 inhibitors (COXIBs), nonselective nonsteroidal anti-inflammatory drugs (NSAIDs) and systemic steroids, the start date, number of pills, dosage, and days supplied were retrieved from the prescription claims and used to construct the daily drug exposure matrix [8]. The daily exposure matrix was then used to calculate time-dependent measures of cumulative duration of use of a specific drug, or class of drugs until a given day during the follow up [9]. For overlapping prescriptions of the same drug, the individual was assumed to have had prescriptions refilled early and completed the first prescription before starting the second. The same rule was applied to overlapping prescriptions of the same drug but with different doses and overlapping prescriptions for different drugs within the same drug class (anti-TNF agents, systemic steroids, COXIBs or NSAIDs). However, given that combination use is common for DMARDs, prescriptions for each class of DMARD were treated separately. When there was a gap of 7 days or less between two prescriptions of the same drug, or of different drugs within the same drug class (excluding DMARDs), it was assumed that the drug was taken continuously and the gap was filled with the daily dose of the second prescription. Exposures to our main anti-rheumatic drugs included 1) MTX and 2) other DMARDs besides MTX.

### Outcomes

The outcome of interest was the time from cohort entry to the first joint replacement surgery (for any joint), defined using the Canadian Classification of Health Intervention (CCI) and the Canadian Classification of Diagnostic, Therapeutic and Surgical Procedures (CCP) procedure codes for joint replacement (see Appendix 1).

### Covariates

Variables that were available in the administrative databases and considered potential confounders for the association between the drugs of interest and joint replacement were selected a priori and adjusted for in all the multivariable models. These included sex, age at cohort entry (in years and with a squared-age term added to account for non-linear effects), place of residence (urban or rural, defined from postal codes) social assistance status at cohort entry, and ecological measures based on census data, on income, education level, and employment rate in the area. We used diagnostic codes from all outpatient physician and/or hospital visits during the 3 years before cohort entry to assess comorbidities (including osteoarthritis (OA), myocardial infarction, diabetes, osteoporosis, cerebrovascular disease, acute renal failure, chronic renal failure, coronary artery disease, chronic obstructive pulmonary disease (COPD), asthma, cancer and the Charlson index). To discriminate between high and low users of the health care system, we adjusted for a binary indicator of high users, defined as people with at least 20 physician visits in at least 1 year of the 3 years before baseline. To adjust for disease severity, we used a time-varying variable capturing the number of rheumatologist visits during the follow-up period (log-transformed). We also controlled for a time-varying indicator of the presence of extra-articular manifestations of the disease during the follow up; this included rheumatoid lung, Felty’s syndrome, rheumatoid carditis, eye involvement, dermatological complications (vasculitis, pyoderma gangrenosum), neuropathies and amyloidosis. Finally, we adjusted for time-dependent variables reflecting cumulative use of other drugs, anti-TNF inhibitors, other biologics, COXIBs, NSAIDs, and systemic steroids during the follow up, and binary indicators of anti-TNF, MTX, other DMARDs, COXIBs, NSAIDs, and systemic steroid use during the period of 1 year before cohort entry.

### Statistical analyses

Descriptive statistics were used to characterize the study population. We used a Cox proportional hazards (PH) regression model with time-dependent variables measuring drug use for 1) MTX and 2) other DMARDs. As mentioned, the model also controlled for concomitant drug exposure (anti-TNF inhibitors, other biologics, COXIBs, NSAIDs, steroids). Our primary analyses considered the effects of early use of MTX and/or DMARDs, that is, in the first year of follow up only. Different time windows for cumulative drug use were considered in alternate models [10]: 1) during the second year of follow up, 2) throughout the entire follow up, and 3) throughout follow up but not including the year prior to the index time for each event. The cumulative use was obtained by summing the duration of all prescriptions for the relevant drugs, up to a given day over the relevant time period. We also tested for an interaction term between the cumulative effects of MTX and other DMARDs to account for possibly increased or decreased risks among users of both drug classes. Adjusted hazard ratios (HRs) with 95 % CIs were generated. The fit to the data of the different models was compared with the Akaike Information Criterion (AIC) [11].

In sensitivity analyses, we weighted the cumulative exposure to evaluate if weighting past exposure by recent use would improve the prediction of joint replacement surgery [12]. This method estimates from the data the relative weights of the timing of past exposure of each of MTX and other DMARDs on risk of outcome. We also performed sensitivity analysis excluding subjects with previous diagnosis of OA or excluding the cases of hip replacements, as these are less likely to be attributed to RA in the early years. Finally in additional sensitivity analysis, we used the propensity scores to adjust for potential differences associated with the exposure of primary interest, i.e., between the characteristics of patients who received different treatments during the first year after the cohort entry. To this end, we first used logistic regression to estimate separate propensity scores (PS) for the first-year treatment with 1) MTX and 2) other DMARDs. Both PS estimated the probability of receiving the respective treatment as a function of all time-fixed characteristics available at the entry into the cohort. In two separate analyses, we then included both PS in the multivariable Cox models that also included the cumulative first-year exposures to 1) MTX, 2) other DMARDs, and 3) their interaction, and all time-dependent potential confounders. The difference between the two PS-adjusted models was that the first also included all time-fixed variables, while the second excluded these time-fixed variables.

## Results

We studied 11,365 incident RA patients who were followed for at least 1 year. Baseline characteristics of the RA patients are shown in Table 1. A majority (67.4 %) were women, with a mean age of 65.3 (standard deviation 14.5) years at cohort entry.

**Table 1:** Baseline characteristics of the incident rheumatoid arthritis cohort

Variable	Number	Percent or SD
Female	7,639	67.4
Age in years at cohort entry	65.3	14.5
Recipient of social assistance at cohort entry^a^	939	8.3
Urban residence^a^	8,884	78.4
High healthcare use at baseline	2981	26.3
Prior joint replacement	352	3.14
Comorbidities		
Myocardial infarction	291	2.6
Diabetes	1,668	14.7
Osteoporosis	1,722	15.2
Cerebrovascular disease	54	0.48
Acute renal failure	314	2.8
Chronic renal failure	298	2.6
Coronary artery disease	1,952	17.2
COPD-asthma	2,587	22.8
Any type of cancer	1,326	11.7
Osteoarthritis	3,469	30.6
Charlson comorbidity	1.2	1.9
Drug use prior to cohort entry		
GC	4,360	38.5
COXIBs	4,824	42.6
NSAIDs	4,491	39.6

*COPD* chronic obstructive pulmonary disease, *GC* glucocorticoids, *COXIBs* cyclooxygenase-2 inhibitors, *NSAIDs* nonselective nonsteroidal anti-inflammatory drugs

^a^Defined by census tract data

A total of 608 joint replacements occurred during 55,780 person-years (median 4.6 years) of follow up, yielding a rate of 1.09 joint replacements per 100 person-years. The knee (41.6 %) and hip (28.2 %) were the most frequent locations for joint replacement. During follow up, 10 % of the patients filled at least one prescription for anti-TNF (ever-users), 58 % for MTX and 65 % had used another DMARD.

**Fig. 1:**
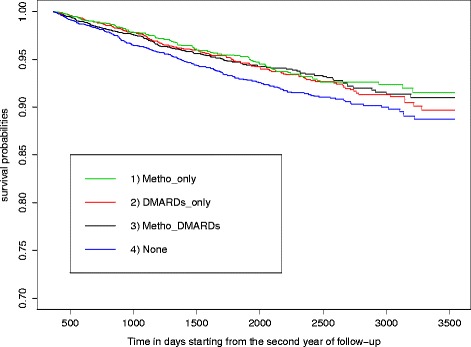
Kaplan-Meier estimates of time to joint replacement surgery. Groups of drug exposure were based on treatment(s) received during the first year after the cohort entry: 1) users of methotrexate (*MTX*) only (*Metho_only*); 2) users of other disease-modifying anti-rheumatic drugs (*DMARD*) only (*DMARDs_only*); 3) users of both MTX and DMARDs (*Metho_DMARDs*); and 4) patients not prescribed either MTX or any other DMARD during the first year of follow up (*None*)

Among the alternative measures of drug exposure, the best-fitting multivariable Cox model relied on the cumulative duration of drug use in the first year after cohort entry (Table 2). Joint replacement throughout follow up was significantly lower for patients with higher cumulative duration of either MTX (HR = 0.95 per month of use, 95 % CI 0.93, 0.97) or of other DMARDs (HR = 0.97 per month of use, 95 % CI 0.95, 0.99) within the first year after cohort entry. These results can respectively be translated into a 5 % decrease in the hazard of surgery associated with every additional month of early MTX use in the first year of follow up, and a 3 % decrease in the hazard of surgery for every additional cumulative month of early use of other DMARDs. These associations became much stronger with prolonged use of the relevant drugs in the first year. For example, the early use of MTX or other DMARDs for 6 months was associated with 31 % (HR = 0.69, 95 % CI 0.59, 0.80) and 27 % (HR = 0.83 95 % CI 0.73, 0.95) reduction in the hazard of joint replacement surgery during follow up. To facilitate assessment of the impact of early treatment with MTX and/or other DMARDs, Fig. 1 compares the Kaplan-Meier-like curves, for the proportions of patients who remain free of joint replacement in four subgroups, defined based on treatment(s) received during the first year after the cohort entry: 1) users of MTX only (*Metho_only*); 2) users of other DMARDs only (*DMARDs_only*); 3) users of both MTX and DMARDs (*Metho_DMARDs*); and 4) patients not prescribed either MTX or any other DMARDs during the first year of follow up (*None*). It is evident that patients treated with MTX and/or other DMARDs in the first year have lower long-term risks of requiring joint replacement, with cumulative risk during the first 4–8 years of follow up lower by about 2 % (Fig. 1). For example, after about 6 years (2,000 days) the cumulative risk is about 5 % in those treated versus about 7 % in those not treated in the first year.

**Table 2:** Best-fitting Cox regression model of time to joint replacement surgery

Variable	Hazard ratio	95 % CI
Age (at cohort entry)	1.19	1.10, 1.28
Age (at cohort entry) squared	1.00	1.00, 1.00
Female sex	0.80	0.66, 0.96
Recipient of social assistance at cohort entry	0.89	0.59, 1.35
Baseline urban residence	1.03	0.82, 1.28
Baseline regional employment rate^a^	1.00	0.98, 1.01
Baseline regional family income^a^	1.00	0.99, 1.01
Baseline regional percent university graduates^a^	1.00	0.99, 1.01
Extra-articular RA manifestations (time-dependent)	1.08	0.91, 1.28
Rheumatology visits at baseline (log-transformed)	1.24	1.11, 1.38
Rheumatology visits in follow up, time dependent (log-transformed)	0.98	0.84, 1.15
High health care use at baseline	1.11	0.91, 1.35
Prior joint replacement	2.55	1.90, 3.43
Baseline comorbidities		
Myocardial infarction	0.86	0.48, 1.56
Diabetes	0.99	0.76, 1.28
Osteoporosis	0.79	0.63, 0.99
Cerebrovascular disease	1.27	0.40, 4.02
Acute renal failure	1.16	0.68, 1.97
Chronic renal failure	1.60	0.90, 2.84
Coronary artery disease	1.01	0.81, 1.27
COPD-asthma	0.96	0.77, 1.18
Any type of cancer	1.01	0.72, 1.41
Osteoarthritis	2.15	1.81, 2.55
Charlson Comorbidity	0.92	0.85, 1.00
Drug use prior to cohort entry		
Anti-TNF	1.00	0.35, 2.85
MTX	1.27	0.94, 1.70
Other DMARDs	1.13	0.88, 1.45
Systemic corticosteroids	1.15	0.97, 1.38
COXIBs	0.97	0.82, 1.15
NSAIDs	0.87	0.73, 1.03
Time-dependent cumulative drugs in follow up (years)		
Anti-TNF	1.13	0.99, 1.29
Other biologic drugs	1.37	0.61, 3.08
Systemic corticosteroids	1.01	0.94, 1.08
COXIBs	1.20	1.11, 1.30
NSAIDs	1.31	1.20, 1.43
Cumulative MTX in first year (months)	0.94	0.92, 0.96
Cumulative other DMARDs in first year months)	0.97	0.95, 0.99

*RA* rheumatoid arthritis, *MTX* methotrexate, *DMARD* disease-modifying anti-rheumatic drug, *COPD* chronic obstructive pulmonary disease, *TNF* tumor necrosis factor, *COXIB* cyclooxygenase-2 inhibitor, *NSAID* nonsteroidal anti-inflammatory drug

^a^Defined by census tract data

The model included an interaction between MTX and other DMARDs: patients who used drugs in both classes during the first year after cohort entry had a significantly shorter time to joint replacement than those who used only one of the two drug classes. We were unable to estimate precisely the effect of cumulative duration of anti-TNF agents during follow up (adjusted HR = 1.13 per year of use, 95 % CI 0.99, 1.29) due to the small number of anti-TNF users. Cumulative duration of NSAIDs and COXIBs were associated with higher risk of joint replacement (NSAIDs: HR = 1.31 per year of use, 95 % CI 1.20, 1.43; COXIBs: HR = 1.20 per year of use, 95 % CI 1.11, 1.30), as were a previous joint replacement (adjusted HR = 2.55, 95 % CI 1.90, 3.43), rheumatology visit at baseline (HR = 1.24, 95 % CI 1.11, 1.38) and diagnosis of OA in the 3 years prior to RA onset (adjusted HR = 2.15, 95 % CI 1.81, 2.55). The sensitivity analyses based on the novel, flexible weighted cumulative exposure model did not improve our estimates of the long-term effects of RA drug exposure. In sensitivity analysis, when we excluded individuals with diagnosis of OA at baseline, the estimates of the cumulative duration of MTX remained similar to our main analysis (HR = 0.92 per month of use, 95 % CI 0.89, 0.96). The effect of MTX was also essentially unchanged by exclusion of the patients with hip replacements (HR = 0.95, 95 % CI 0.91, 0.99). However, in both sensitivity analyses, the confidence interval was widened for the effect of other DMARDs. In the PS-adjusted models, the estimated effects of both main exposures, and of their interaction, were very similar to the estimates from our multivariable model (see Additional file 1).

## Discussion

In our sample of patients with new-onset RA, increased duration of exposure to either MTX or other DMARDs early after the cohort entry was associated with significantly longer time to joint replacement. This finding is consistent with other studies suggesting that early use of DMARDs results in both short-term and long-term benefits [13–16]. In a population-based cohort study, patients treated early with DMARDs (within the first year of symptom onset) had a significantly lower progression rate in RA compared with patients who initiated treatment 1–5 years after symptom onset [15]. Results from the Utrecht Rheumatoid Arthritis Cohort showed that treatment with conventional DMARDs immediately after diagnosis resulted in less joint surgery when compared with a delayed start [16]. The rationale for a prompt initiation of DMARDs in patients with RA is based on the idea that there is a critical therapeutic window of opportunity, in which disease modification is more successful, possibly because the pathogenetic mechanisms are still in early development [3]. Although biologic therapies were introduced to the Quebec formulary in 2002, our study was unable to make any definitive conclusions about a potential association between these therapies and later need for joint surgery. In the current dataset, very early use (within the first year after RA onset) of biologics was rare.

A caveat to our main findings is that among the cohort members with incident RA, about a quarter did not receive any DMARD therapy during the follow-up time (median of 4.5 years). These individuals tended to be older, and to have more comorbidities at baseline. This combination of increased age and comorbidity may represent a relative contraindication to DMARD use in this subgroup. Conversely, they may represent a subgroup with milder disease. We lack the clinical data to make this determination. This group also had a higher frequency of pre-existing OA and joint replacement surgery even prior to the cohort entry, and therefore would be expected to be at high risk of requiring future joint surgery. This subset of individuals not receiving MTX or other DMARDs, being at high risk of requiring future joint surgery for OA, could explain part of the observed potentially protective effect of cumulative MTX and other DMARD use in the first year of cohort entry. Although the primary indication for joint surgery could not be confirmed from the data, we did seek to address this issue by adjusting for baseline presence of OA and for current and past exposure to NSAIDs and COXIBs.

In our study, patients who were concurrently or sequentially prescribed both MTX and other DMARDs within the first year after cohort entry had a shorter time to joint replacement, i.e., a higher risk of joint replacement, than users of only one of these drug classes. This perhaps reflects unmeasured confounding by indication, if the use of combination DMARDs is taken as a marker for patients with the most active or severe disease, and accordingly those who are the most susceptible to need early surgery. Confounding by indication has been demonstrated in other observational RA cohorts, with appropriate adjustments made during analysis [17, 18]. While administrative data have the advantage of being population-based and including large numbers of patients, such data do not include the clinical parameters needed to adjust for disease severity/potential confounding by indication.

Given the observational nature of our study, our results are also subject to other possible unmeasured confounders and selection bias. For example, it is possible that receiving either MTX alone or other traditional DMARDs is a marker of unmeasured patient characteristics that would influence willingness to receive joint replacement surgery. Other factors that may be effect modifiers, such as smoking or body mass index, were unavailable in our dataset, although it is unclear how important such variables might be for the current analyses.

Our analyses complement the existing literature. A few studies have investigated whether patients with new-onset RA during the pre-biologic era were more likely to need joint surgery later on, compared to patients with new-onset RA in more recent times (biologics having being available in most developed nations for over a decade) [19, 20]. One, based on chart review in adults with new-onset RA in the US showed that for the period 1980–1994, 12.1 % of patients had undergone joint surgery 10 years after RA onset. In contrast, the authors found that in a similar, more recent retrospective cohort of new-onset RA, followed from 1995–2007, only 6.0 % had had joint surgery after 10 years. The authors interpreted the lower joint replacement rate as indicative of improved RA treatments [19]. Similar conclusions were reached in a Finnish study [21]. Conversely, a multicenter study in Japan did not observe a decrease in the rates of RA-associated orthopedic surgery from 1998–2008, despite a steady increase in the proportion of patients using biologics from 2004 (1.8 %) to 2007 (10.0 %) [22]. One possible explanation for that finding is that in more recent years, patients who seek greater levels of activity may be undergoing joint surgery earlier than patients in the past. In addition, the study from Japan included not just joint replacements but also other types of surgery. Finally, technological advancements mean that patients today have more joint-preserving surgical options than in the past.

The strengths of our study are its population-based nature and the availability of detailed drug prescription data. However, data from administrative sources lack primary clinical information on important clinical characteristics, which makes it difficult to adjust for RA severity. Similar to other database studies of drugs use and their effects in RA [23, 24], we did use proxies of RA severity, including extra-articular manifestations of RA, number of rheumatology visits, and use of concomitant medications like glucocorticoids. This approach has been used by other authors and appears to control to some extent for disease severity [25], though it is not ideal.

Time until joint replacement, as measured in our study, is influenced not only by necessity, but also by healthcare access. For example, individuals with higher socioeconomic status tend to have better access to care, and thus, have shorter waiting times even within publicly funded healthcare systems, to receive elective surgery [26]. In our study, we adjusted for variables related to socioeconomic status, such as being on social assistance, and ecological measures such as family income, employment, and education [27]. We were not able to address other issues related to accessibility, such as wait times to either consult a surgeon or obtain surgery or difficulties on the part of patients to attend appointments. Finally, we have to take some aspects into account when extrapolating our results. The rate of biologic use represents an average over time and accounts for periods when these drugs were not yet widely available. Nevertheless, the frequency of use of both biologics and non-biologic DMARDs found in our study is not greatly different from those documented in similar studies using administrative databases [28]. Moreover, the access to anti-TNF agents in Canada is comparable to that observed in countries with a similar income, with the exception of the USA [29]. All things considered, we believe the results of this study can be informative for other countries with similar sociodemographics characteristics and healthcare access.

## Conclusions

In summary, our study suggests that higher cumulative use of MTX and higher cumulative use of non-MTX traditional DMARDs within the first year after the cohort entry may be associated with longer time to joint surgery. A causal link cannot be firmly established with these observational data; however, these real-world findings may be consistent with joint-sparing effects suggested by other data. Given that biologic therapies were only recently introduced to the Quebec formulary, our study was unable to make any definitive conclusions about the potential effect of these novel therapies and later need for surgery, but analyses of future data using similar methods should investigate this issue.

## Appendix 1

**ᅟ:** List of CCI/CCP procedure codes for joint replacement

Canadian Classification of Health Interventions	
*Code*	*Description*
1EL53	Implantation of internal device, temporomandibular joint [TMJ]
1SQ53	Implantation of device, pelvis
1TA53	Implantation of internal device, shoulder joint
1TM53	Implantation of internal device, elbow joint
1UB53	Implantation of internal device, wrist joint
1UC53	Implantation of internal device, distal radioulnar joint and carpal joints and bones
1UG53	Implantation of internal device, metacarpophalangeal joint(s)
1UH53	Implantation of internal device, first metacarpophalangeal joint
1UK53	Implantation of internal device, interphalangeal joints of hand
1UM53	Implantation of internal device, first interphalangeal joint of hand
1VA53	Implantation of internal device, hip joint
1VG53	Implantation of internal device, knee joint
1VP53	Implantation of internal device, patella
1WA53	Implantation of internal device, ankle joint
1WE53	Implantation of internal device, tarsal bones and intertarsal joints [hindfoot, midfoot]
1WI53	Implantation of internal device, first metatarsal bone and first metatarsophalangeal joint
1WJ53	Implantation of internal device, tarsometatarsal joints, metatarsal bones and metatatarsophalangeal joints [forefoot]
1WM53	Implantation of internal device, interphalangeal joints of toe
1WN53	Implantation of internal device, first interphalangeal joint of toe
Canadian Classification of Diagnostic, Therapeutic and Surgical Procedures (CCP)	
*Code*	*Description*
9341	Total knee replacement (geomedic) (polyc
9348	Total ankle replacement
9350	Total hip replacement
9351	Total hip replacement with methyl methacrylate
9359	Other total hip replacement
9360	Other arthroplasty of hip
9361	Replacement of head of femur with use of methyl methacrylate
9362	Other replacement of head of femur
9363	Replacement of acetabulum with use of methyl methacrylate
9364	Other replacement of acetabulum
9370	Arthroplasty of hand and finger
9371	Arthroplasty of hand and finger with synthetic prosthesis
9380	Arthroplasty of upper extremity, except hand
9381	Arthroplasty of shoulder with synthetic
9384	Arthroplasty of elbow with synthetic prosthesis
9386	Arthroplasty of carpals with synthetic prosthesis

## Additional file

Additional file 1:
**Propensity score analysis.** Results of the propensity score analysis for the main exposure drug groups and comparison with the best-fitting multivariable Cox model. (DOC 39 kb)

## Abbreviations

AIC: Akaike Information Criterion
anti-TNF: anti-tumor necrosis factor
CCI: Canadian Classification of Health Intervention
CCP: Canadian Classification of Diagnostic, Therapeutic and Surgical Procedures
COPD: chronic obstructive pulmonary disease
COXIBs: cyclooxygenase-2 inhibitors
DMARDs: disease-modifying anti-rheumatic drugs
HR: hazard ratio
ICD: International Classification of Diseases
MTX: methotrexate
NSAIDs: nonselective nonsteroidal anti-inflammatory drugs
OA: osteoarthritis
PH: proportional hazards
PS: propensity scores
RA: rheumatoid arthritis
RAMQ: Quebec Health Insurance Program
