# Supplementary material for: Early medication use in new-onset rheumatoid arthritis may delay joint replacement: results of a large population-based study
Source: Arthritis Res Ther. 2015 Aug 3;17(1):197. doi: 10.1186/s13075-015-0713-3 (PMC4522999; doi:10.1186/s13075-015-0713-3)
Supplement: Additional file 1: — Propensity score analysis. Results of the propensity score analysis for the main exposure drug groups and comparison with the best-fitting multivariable Cox model. (DOC 39 kb) [file 13075_2015_713_MOESM1_ESM.doc]

**Additional File 1**

**Table 3-** Results of the propensity score analysis for the main exposure drug groups and comparison with the best-fitting multivariable Cox model.

| Model | HR (95% CI) | | |
| --- | --- | --- | --- |
|  | MTX use in 1^st^ year | Other DMARD | MTX*Other DMARDs Interaction |
| Cox model adjusted for individual covariates | 0.94 (0.92-0.96) | 0.97 (0.95-0.99) | 1.00 (1.00-1.01) |
| PS 1 | 0.95 (0.92-0.97) | 0.97 (0.95-1.00) | 1.00 (1.00-1.01) |
| PS 2 | 0.94 (0,92-0,96) | 0.97 (0.95-0.99) | 1.00 (1.00-1.01) |
| HR: Hazard Ratio; 95% CI : 95% confidence interval  PS 1: Model adjusted for propensity score only  PS 2: Model adjusted for both individual covariates and propensity score  MTX : methotrexate; DMARDs: disease-modifying anti-rheumatic drugs | | | |
